# [2,6-Bis(biphenyl-4-yl)-4-hy­droxy-4-(pyridin-2-yl)cyclo­hexane-1,3-di­yl]bis­[(pyridin-2-yl)methanone]–butan-2-one (1/1)

**DOI:** 10.1107/S1600536812019241

**Published:** 2012-05-05

**Authors:** Hoong-Kun Fun, Chin Wei Ooi, S. Samshuddin, B. Narayana, B. K. Sarojini

**Affiliations:** a X-ray Crystallography Unit, School of Physics, Universiti Sains Malaysia, 11800 USM, Penang, Malaysia; b Department of Studies in Chemistry, Mangalore University, Mangalagangotri 574 199, India; c Department of Chemistry, P.A. College of Engineering, Nadupadavu, Mangalore 574 153, India

## Abstract

In the title solvate, C_47_H_37_N_3_O_3_·C_4_H_8_O, the cyclo­hexane ring adopts a chair conformation and the plane through its near coplanar atoms forms dihedral angles of 82.58 (7), 89.27 (7), 60.30 (8), 54.54 (7) and 72.03 (7)°, respectively, with the three pyridine rings and the two attached benzene rings. The rings of the biphenyl units are twisted from each other, making dihedral angles of 35.27 (7) and 45.41 (7)°. All the rings are in equatorial orientations in the cyclo­hexane ring, except for the C=O-bonded pyridine ring in position 1, which is axial. Intra­molecular O—H⋯N and C—H⋯O hydrogen bonds form one *S*(5) and three *S*(6) ring motifs. In the crystal, mol­ecules are linked *via* C—H⋯O hydrogen bonds into a chain along the *c* axis. The crystal structure also features weak C—H⋯π inter­actions and aromatic π–π stacking [centroid–centroid distances = 3.5856 (10) and 3.7090 (9) Å].

## Related literature

For a related structure, see: Schormann & Egert (1996 ▶). For ring conformations, see: Cremer & Pople (1975 ▶). For the stability of the temperature controller used in the data collection, see: Cosier & Glazer (1986 ▶). For standard bond lengths, see: Allen *et al.* (1987 ▶). For hydrogen-bond motifs, see: Bernstein *et al.* (1995 ▶).
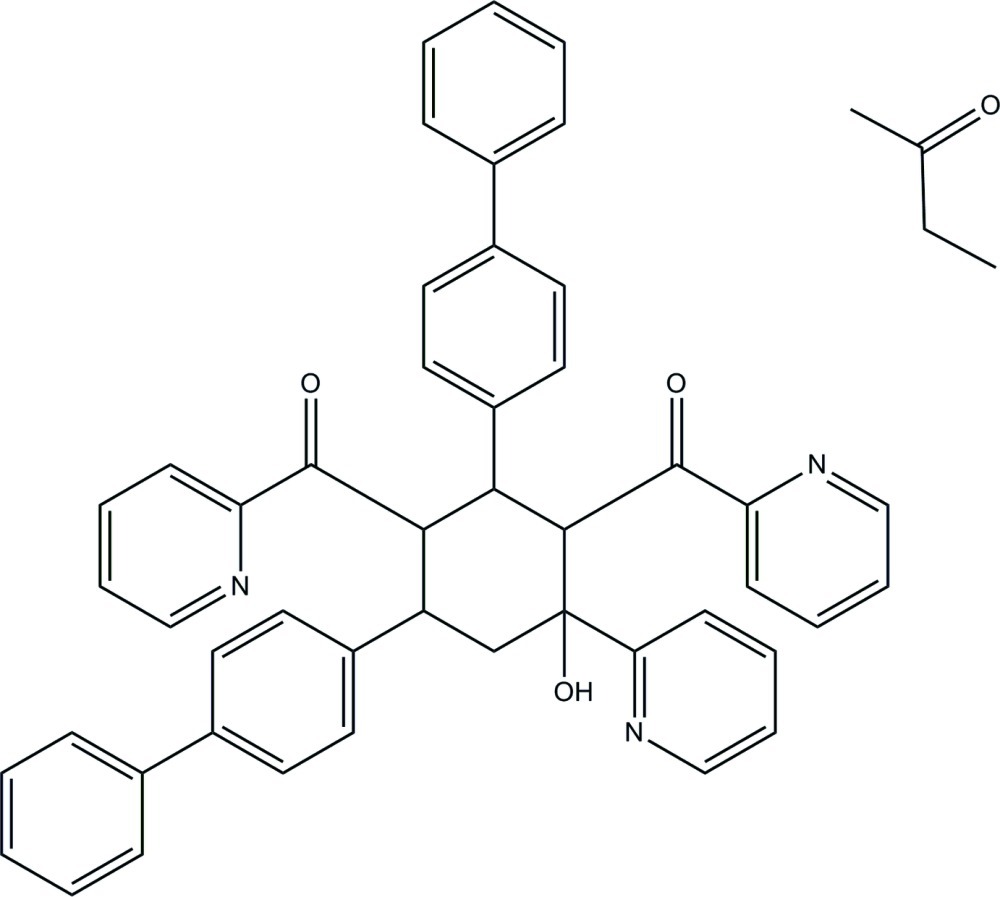


## Experimental

### 

#### Crystal data

C_47_H_37_N_3_O_3_·C_4_H_8_O
*M* *_r_* = 763.90
Monoclinic, 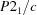

*a* = 13.7269 (9) Å
*b* = 25.3723 (16) Å
*c* = 11.4574 (7) Å
β = 100.643 (1)°
*V* = 3921.8 (4) Å^3^
*Z* = 4
Mo *K*α radiation
μ = 0.08 mm^−1^
*T* = 100 K
0.38 × 0.22 × 0.17 mm

#### Data collection

Bruker APEX DUO CCD diffractometer
Absorption correction: multi-scan (*SADABS*; Bruker, 2009 ▶) *T*_min_ = 0.969, *T*_max_ = 0.987
41041 measured reflections
10584 independent reflections
7594 reflections with *I* > 2σ(*I*)
*R*_int_ = 0.050

#### Refinement

*R*[*F* ^2^ > 2σ(*F* ^2^)] = 0.049
*wR*(*F* ^2^) = 0.143
*S* = 0.99
10584 reflections
529 parameters
H atoms treated by a mixture of independent and constrained refinement
Δρ_max_ = 0.44 e Å^−3^
Δρ_min_ = −0.30 e Å^−3^

### 

Data collection: *APEX2* (Bruker, 2009 ▶); cell refinement: *SAINT* (Bruker, 2009 ▶); data reduction: *SAINT*; program(s) used to solve structure: *SHELXTL* (Sheldrick, 2008 ▶); program(s) used to refine structure: *SHELXTL*; molecular graphics: *SHELXTL*; software used to prepare material for publication: *SHELXTL* and *PLATON* (Spek, 2009 ▶).

## Supplementary Material

Crystal structure: contains datablock(s) global, I. DOI: 10.1107/S1600536812019241/hb6756sup1.cif

Structure factors: contains datablock(s) I. DOI: 10.1107/S1600536812019241/hb6756Isup2.hkl

Supplementary material file. DOI: 10.1107/S1600536812019241/hb6756Isup3.cml

Additional supplementary materials:  crystallographic information; 3D view; checkCIF report

## Figures and Tables

**Table 1:** Hydrogen-bond geometry (Å, °) *Cg*2 is the centroid of the N2/C20–C24 ring.

*D*—H⋯*A*	*D*—H	H⋯*A*	*D*⋯*A*	*D*—H⋯*A*
O1—H1*O*1⋯N1	0.90 (2)	1.92 (2)	2.5615 (18)	127.0 (19)
C16—H16*A*⋯O3	0.97	2.46	3.0730 (19)	121
C18—H18*A*⋯O3	0.98	2.39	3.0603 (19)	125
C26—H26*A*⋯O1^i^	0.93	2.53	3.3079 (19)	142
C27—H27*A*⋯O4^ii^	0.93	2.56	3.464 (2)	163
C3—H3*A*⋯*Cg*2^iii^	0.93	2.96	3.7821 (17)	148

Symmetry codes: (i) 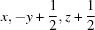; (ii) 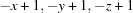; (iii) 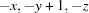.

**Fig. 1.**
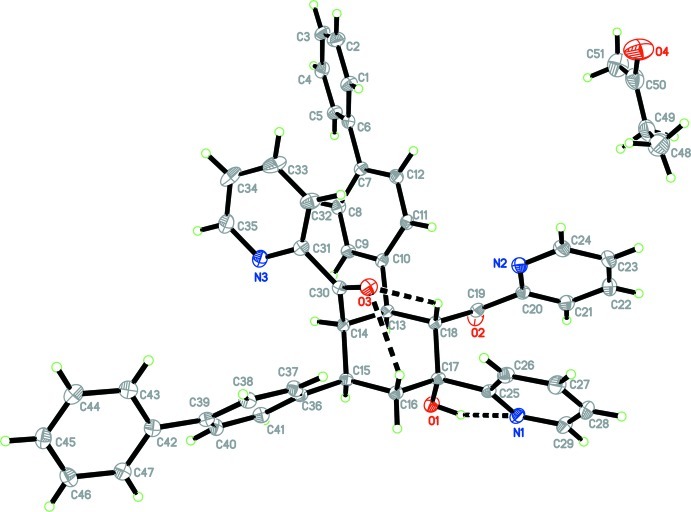
The molecular structure of the title compound, showing 50% probability displacement ellipsoids. Intramolecular hydrogen bonds are shown as dashed lines.

**Fig. 2.**
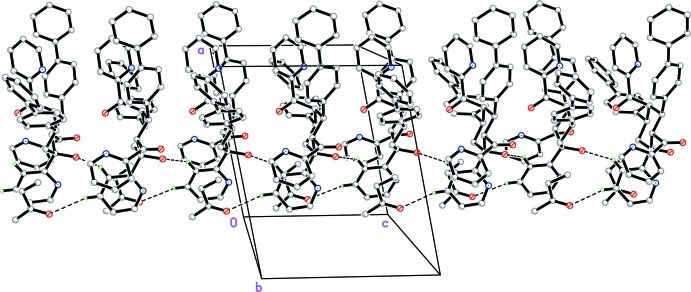
The crystal packing of the title compound, viewed along the b axis. H atoms not involved in the intermolecular interactions (dashed lines) have been omitted for clarity.

**Fig. 3.**
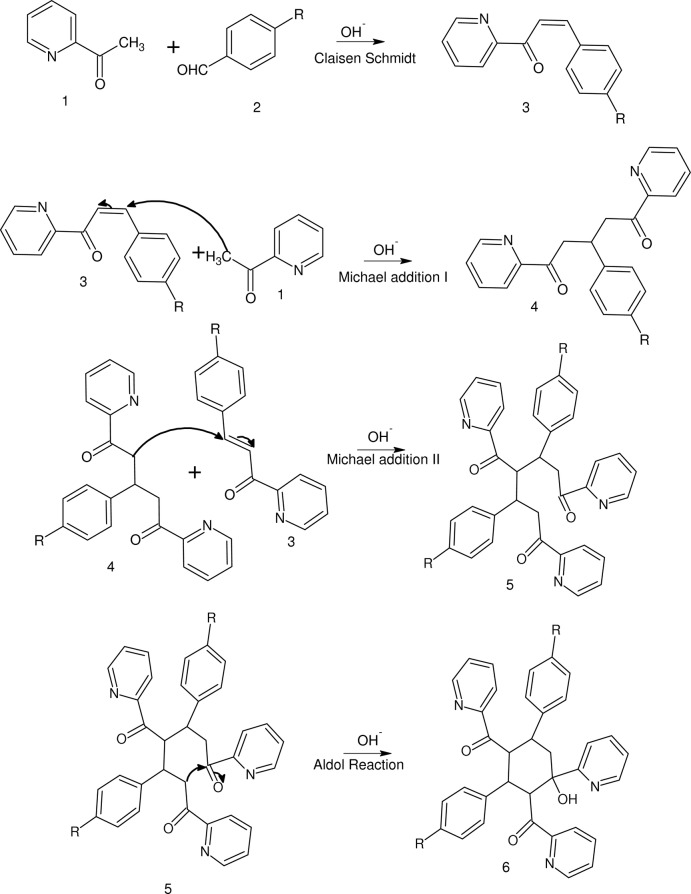
Proposed mechanism of formation of the title compound. R = C6H5.

## supplementary crystallographic information

### Comment

The crystal structure of fully *cyclo*-substituted cyclohexane, 1*r*,9*t*,16*t*-trioxahexaspiro[2.0.3.0.2.0.3.0.2.0.3.0]heneicosane, has been reported (Schormann & Egert, 1996). As part of our studies in this area, the title compound (I) was prepared and its crystal structure is now reported.

The central cyclohexane (C13–C18) ring adopts a chair conformation (Cremer & Pople, 1975) with puckering parameters Q=0.5932 (16) Å, θ=4.69 (15)°, φ=274.6 (19)° and the plane through the coplanar atoms (C13/C15/C16/C18) form dihedral angles of 82.58 (7), 89.27 (7), 60.30 (8), 54.54 (7), and 72.03 (7)° repectively with three pyridines (N1/C25–C29, N2/C20–C24 and N3/C31–C35) rings and two attached phenyl (C7–C12 and C36–C41) rings. The phenyl rings of biphenyl (C1–C6/C7–C12 and C36–C41/C42–C47) are slightly twisted from each other with the dihedral angles of 35.27 (7) and 45.41 (7)°, respectively. Intramolecular O1—H1O1···N1, C16—H16A···O3 and C18—H18A···O3 hydrogen bonds (Table 1) form one S(5) and three S(6) ring motifs (Bernstein *et al.*, 1995). The bond lengths are comparable to those in the related structure (Schormann & Egert, 1996).

In the crystal (Fig. 2), the molecules are linked *via* intermolecular C26—H26A···O1 and C27—H27A···O4 hydrogen bonds into a chain along the *c* axis. The crystal structure is further stabilized by C—H···π interactions (Table 1), involving the N2/C20–C24 ring (centroid *Cg*2). Weak π—π interactions were observed with *Cg*1···*Cg*2 = 3.5856 (10) Å [symmetry code: *X*, Y, *Z*] and *Cg*8···*Cg*8 = 3.7090 (9) Å [symmetry code: –*X*, –Y, –*Z*], where *Cg*1, *Cg*2 and *Cg*8 are the centroids of N1/C25–C29, N2/C20–C24 and C42–C47 rings, respectively.

### Experimental

To a mixture of 2-acetyl pyridine (0.363 g, 0.003 mole) and biphenyl-4-carboxaldehyde (0.42 g, 0.002 mole) in 30 ml ethanol, 10 ml of 10% potassium hydroxide solution was added and stirred at 15–20 °C for 4 h and kept at room temperature overnight. The precipitate formed was collected by filtration and purified by recrystallization from acetone. The proposed mechanism is shown in Fig. 3. Colourless blocks were grown from EMK by the slow evaporation method and the yield of the compound was 74%. (*M.p.*: 518 K).

### Refinement

Atom H1O1 was located from the difference map and refined freely [O—H = 0.90 (2) Å]. The remaining H atoms were positioned geometrically and refined using a riding model with *U*_iso_(H) = 1.2 or 1.5 *U*_eq_(C) (C—H = 0.9300–0.9800 Å). A rotating group model was applied to the methyl groups. In the final refinement, 4 outliers (0 10 0), (7 1 5), (-8 1 6) and (6 1 0) were omitted.

### Crystal data

**Table:**

C_47_H_37_N_3_O_3_·C_4_H_8_O	*F*(000) = 1616
*M**_r_* = 763.90	*D*_x_ = 1.294 Mg m^−^^3^
Monoclinic, *P*2_1_/*c*	Mo *K*α radiation, λ = 0.71073 Å
Hall symbol: -P 2ybc	Cell parameters from 7618 reflections
*a* = 13.7269 (9) Å	θ = 2.2–28.6°
*b* = 25.3723 (16) Å	µ = 0.08 mm^−^^1^
*c* = 11.4574 (7) Å	*T* = 100 K
β = 100.643 (1)°	Block, colourless
*V* = 3921.8 (4) Å^3^	0.38 × 0.22 × 0.17 mm
*Z* = 4	

### Data collection

**Table:**

Bruker APEX DUO CCD diffractometer	10584 independent reflections
Radiation source: fine-focus sealed tube	7594 reflections with *I* > 2σ(*I*)
Graphite monochromator	*R*_int_ = 0.050
φ and ω scans	θ_max_ = 29.2°, θ_min_ = 1.5°
Absorption correction: multi-scan (*SADABS*; Bruker, 2009)	*h* = −18→18
*T*_min_ = 0.969, *T*_max_ = 0.987	*k* = −34→34
41041 measured reflections	*l* = −15→15

### Refinement

**Table:**

Refinement on *F*^2^	Primary atom site location: structure-invariant direct methods
Least-squares matrix: full	Secondary atom site location: difference Fourier map
*R*[*F*^2^ > 2σ(*F*^2^)] = 0.049	Hydrogen site location: inferred from neighbouring sites
*wR*(*F*^2^) = 0.143	H atoms treated by a mixture of independent and constrained refinement
*S* = 0.99	*w* = 1/[σ^2^(*F*_o_^2^) + (0.0773*P*)^2^ + 1.0792*P*] where *P* = (*F*_o_^2^ + 2*F*_c_^2^)/3
10584 reflections	(Δ/σ)_max_ = 0.001
529 parameters	Δρ_max_ = 0.44 e Å^−^^3^
0 restraints	Δρ_min_ = −0.30 e Å^−^^3^

### Special details

**Table:**

Experimental. The crystal was placed in the cold stream of an Oxford Cryosystems Cobra open-flow nitrogen cryostat (Cosier & Glazer, 1986) operating at 100.0 (1) K.
Geometry. All e.s.d.'s (except the e.s.d. in the dihedral angle between two l.s. planes) are estimated using the full covariance matrix. The cell e.s.d.'s are taken into account individually in the estimation of e.s.d.'s in distances, angles and torsion angles; correlations between e.s.d.'s in cell parameters are only used when they are defined by crystal symmetry. An approximate (isotropic) treatment of cell e.s.d.'s is used for estimating e.s.d.'s involving l.s. planes.
Refinement. Refinement of *F*^2^ against ALL reflections. The weighted *R*-factor *wR* and goodness of fit *S* are based on *F*^2^, conventional *R*-factors *R* are based on *F*, with *F* set to zero for negative *F*^2^. The threshold expression of *F*^2^ > σ(*F*^2^) is used only for calculating *R*-factors(gt) *etc*. and is not relevant to the choice of reflections for refinement. *R*-factors based on *F*^2^ are statistically about twice as large as those based on *F*, and *R*- factors based on ALL data will be even larger.

### Fractional atomic coordinates and isotropic or equivalent isotropic displacement parameters (Å^2^)

**Table:**

	*x*	*y*	*z*	*U*_iso_*/*U*_eq_	
O1	0.47599 (8)	0.28118 (4)	−0.07705 (9)	0.0183 (2)	
O2	0.40319 (8)	0.39570 (5)	−0.08531 (10)	0.0238 (3)	
O3	0.25110 (8)	0.26318 (4)	0.19706 (9)	0.0186 (2)	
N1	0.62179 (9)	0.32082 (5)	0.06856 (12)	0.0194 (3)	
N2	0.41942 (9)	0.40787 (5)	0.22129 (12)	0.0190 (3)	
N3	0.03605 (9)	0.22770 (5)	−0.00083 (12)	0.0202 (3)	
C1	−0.11669 (11)	0.44153 (7)	0.03464 (14)	0.0210 (3)	
H1A	−0.0799	0.4296	0.1062	0.025*	
C2	−0.20671 (12)	0.46671 (7)	0.03377 (16)	0.0247 (3)	
H2A	−0.2293	0.4719	0.1046	0.030*	
C3	−0.26301 (11)	0.48415 (6)	−0.07207 (16)	0.0229 (3)	
H3A	−0.3236	0.5007	−0.0727	0.027*	
C4	−0.22829 (11)	0.47679 (6)	−0.17722 (15)	0.0215 (3)	
H4A	−0.2658	0.4885	−0.2485	0.026*	
C5	−0.13774 (11)	0.45199 (6)	−0.17696 (14)	0.0193 (3)	
H5A	−0.1151	0.4475	−0.2479	0.023*	
C6	−0.08069 (10)	0.43384 (6)	−0.07068 (13)	0.0164 (3)	
C7	0.01449 (10)	0.40547 (6)	−0.06783 (13)	0.0154 (3)	
C8	0.02761 (11)	0.37150 (6)	−0.15985 (13)	0.0172 (3)	
H8A	−0.0223	0.3682	−0.2264	0.021*	
C9	0.11430 (11)	0.34263 (6)	−0.15275 (13)	0.0161 (3)	
H9A	0.1217	0.3203	−0.2150	0.019*	
C10	0.19083 (10)	0.34639 (6)	−0.05390 (13)	0.0136 (3)	
C11	0.17924 (10)	0.38177 (6)	0.03568 (13)	0.0155 (3)	
H11A	0.2301	0.3860	0.1009	0.019*	
C12	0.09242 (11)	0.41081 (6)	0.02849 (13)	0.0166 (3)	
H12A	0.0862	0.4342	0.0891	0.020*	
C13	0.27781 (10)	0.30855 (6)	−0.04452 (12)	0.0136 (3)	
H13A	0.2959	0.3064	−0.1232	0.016*	
C14	0.24354 (10)	0.25255 (6)	−0.01356 (12)	0.0131 (3)	
H14A	0.1902	0.2416	−0.0781	0.016*	
C15	0.32949 (10)	0.21228 (6)	−0.00748 (13)	0.0138 (3)	
H15A	0.3457	0.2111	−0.0872	0.017*	
C16	0.42367 (10)	0.23047 (6)	0.07589 (13)	0.0155 (3)	
H16A	0.4115	0.2318	0.1566	0.019*	
H16B	0.4763	0.2052	0.0736	0.019*	
C17	0.45672 (10)	0.28472 (6)	0.04085 (13)	0.0142 (3)	
C18	0.37070 (10)	0.32549 (6)	0.04325 (13)	0.0134 (3)	
H18A	0.3551	0.3260	0.1233	0.016*	
C19	0.40692 (10)	0.37974 (6)	0.01556 (13)	0.0163 (3)	
C20	0.45398 (10)	0.41334 (6)	0.11962 (14)	0.0166 (3)	
C21	0.52927 (11)	0.44845 (6)	0.10656 (16)	0.0222 (3)	
H21A	0.5502	0.4517	0.0342	0.027*	
C22	0.57213 (12)	0.47830 (7)	0.20346 (17)	0.0287 (4)	
H22A	0.6227	0.5020	0.1976	0.034*	
C23	0.53843 (12)	0.47235 (7)	0.30941 (17)	0.0278 (4)	
H23A	0.5672	0.4912	0.3765	0.033*	
C24	0.46085 (12)	0.43761 (7)	0.31349 (15)	0.0236 (3)	
H24A	0.4366	0.4349	0.3838	0.028*	
C25	0.55011 (10)	0.30321 (6)	0.12396 (13)	0.0156 (3)	
C27	0.64309 (12)	0.32676 (7)	0.31461 (15)	0.0246 (3)	
H27A	0.6507	0.3284	0.3969	0.030*	
C26	0.55883 (11)	0.30471 (6)	0.24721 (14)	0.0190 (3)	
H26A	0.5089	0.2911	0.2834	0.023*	
C28	0.71596 (12)	0.34637 (7)	0.25710 (16)	0.0268 (4)	
H28A	0.7725	0.3622	0.3000	0.032*	
C29	0.70311 (11)	0.34191 (7)	0.13515 (16)	0.0244 (3)	
H29A	0.7532	0.3541	0.0974	0.029*	
C30	0.19993 (10)	0.25650 (6)	0.09887 (13)	0.0141 (3)	
C31	0.08895 (10)	0.25547 (6)	0.08853 (13)	0.0159 (3)	
C32	0.04766 (12)	0.28204 (7)	0.17401 (14)	0.0228 (3)	
H32A	0.0874	0.3006	0.2348	0.027*	
C33	−0.05412 (12)	0.28006 (8)	0.16618 (16)	0.0303 (4)	
H33A	−0.0844	0.2982	0.2204	0.036*	
C34	−0.11000 (12)	0.25067 (7)	0.07614 (16)	0.0288 (4)	
H34A	−0.1784	0.2480	0.0696	0.035*	
C35	−0.06176 (12)	0.22533 (7)	−0.00385 (16)	0.0248 (3)	
H35A	−0.0998	0.2054	−0.0635	0.030*	
C36	0.29503 (10)	0.15738 (6)	0.01718 (13)	0.0145 (3)	
C37	0.29334 (11)	0.13891 (6)	0.13140 (13)	0.0171 (3)	
H37A	0.3198	0.1596	0.1966	0.020*	
C38	0.25277 (11)	0.09003 (6)	0.14942 (13)	0.0175 (3)	
H38A	0.2509	0.0790	0.2263	0.021*	
C39	0.21496 (10)	0.05740 (6)	0.05408 (13)	0.0159 (3)	
C40	0.22118 (11)	0.07492 (6)	−0.06035 (13)	0.0178 (3)	
H40A	0.1991	0.0532	−0.1252	0.021*	
C41	0.25965 (11)	0.12404 (6)	−0.07825 (13)	0.0167 (3)	
H41A	0.2620	0.1350	−0.1551	0.020*	
C42	0.16365 (11)	0.00691 (6)	0.06980 (13)	0.0171 (3)	
C43	0.09407 (11)	0.00461 (6)	0.14501 (14)	0.0198 (3)	
H43A	0.0858	0.0335	0.1922	0.024*	
C44	0.03753 (11)	−0.04023 (7)	0.14967 (14)	0.0217 (3)	
H44A	−0.0091	−0.0409	0.1991	0.026*	
C45	0.04944 (11)	−0.08409 (6)	0.08162 (15)	0.0215 (3)	
H45A	0.0101	−0.1138	0.0839	0.026*	
C46	0.12103 (12)	−0.08316 (6)	0.00972 (14)	0.0212 (3)	
H46A	0.1310	−0.1127	−0.0347	0.025*	
C47	0.17744 (11)	−0.03803 (6)	0.00448 (13)	0.0183 (3)	
H47A	0.2252	−0.0378	−0.0434	0.022*	
O4	0.27860 (12)	0.65693 (7)	0.38250 (13)	0.0479 (4)	
C49	0.39719 (14)	0.63104 (8)	0.26608 (18)	0.0345 (4)	
H49A	0.4438	0.6575	0.2497	0.041*	
H49B	0.3815	0.6086	0.1967	0.041*	
C50	0.30448 (14)	0.65787 (8)	0.28538 (17)	0.0319 (4)	
C51	0.24465 (16)	0.68586 (9)	0.18082 (18)	0.0387 (5)	
H51A	0.1956	0.7075	0.2071	0.058*	
H51B	0.2125	0.6604	0.1246	0.058*	
H51C	0.2876	0.7075	0.1439	0.058*	
C48	0.44650 (15)	0.59802 (8)	0.3706 (2)	0.0419 (5)	
H48A	0.5091	0.5853	0.3562	0.063*	
H48B	0.4047	0.5686	0.3805	0.063*	
H48C	0.4569	0.6192	0.4413	0.063*	
H1O1	0.5333 (17)	0.2988 (9)	−0.074 (2)	0.042 (6)*	

### Atomic displacement parameters (Å^2^)

**Table:**

	*U*^11^	*U*^22^	*U*^33^	*U*^12^	*U*^13^	*U*^23^
O1	0.0171 (5)	0.0259 (6)	0.0128 (5)	−0.0020 (4)	0.0055 (4)	−0.0020 (4)
O2	0.0266 (6)	0.0269 (6)	0.0167 (6)	−0.0052 (5)	0.0010 (5)	0.0061 (5)
O3	0.0196 (5)	0.0237 (6)	0.0123 (5)	0.0008 (4)	0.0020 (4)	0.0003 (4)
N1	0.0142 (6)	0.0228 (7)	0.0216 (7)	−0.0008 (5)	0.0042 (5)	−0.0014 (5)
N2	0.0181 (6)	0.0206 (7)	0.0178 (6)	0.0002 (5)	0.0017 (5)	−0.0016 (5)
N3	0.0175 (6)	0.0206 (7)	0.0222 (7)	−0.0021 (5)	0.0032 (5)	0.0012 (5)
C1	0.0194 (7)	0.0256 (8)	0.0181 (8)	0.0013 (6)	0.0038 (6)	0.0006 (6)
C2	0.0231 (8)	0.0275 (9)	0.0257 (9)	0.0016 (6)	0.0105 (7)	−0.0008 (7)
C3	0.0147 (7)	0.0200 (8)	0.0344 (9)	0.0021 (6)	0.0060 (6)	0.0018 (7)
C4	0.0169 (7)	0.0206 (8)	0.0257 (8)	0.0005 (6)	0.0003 (6)	0.0051 (6)
C5	0.0180 (7)	0.0208 (8)	0.0191 (8)	0.0010 (6)	0.0032 (6)	0.0012 (6)
C6	0.0152 (6)	0.0162 (7)	0.0173 (7)	−0.0012 (5)	0.0022 (6)	−0.0006 (6)
C7	0.0147 (6)	0.0169 (7)	0.0147 (7)	0.0002 (5)	0.0032 (5)	0.0025 (6)
C8	0.0164 (7)	0.0216 (8)	0.0123 (7)	0.0006 (5)	−0.0008 (5)	0.0005 (6)
C9	0.0175 (7)	0.0199 (7)	0.0107 (7)	0.0009 (5)	0.0019 (5)	−0.0021 (6)
C10	0.0131 (6)	0.0147 (7)	0.0129 (7)	−0.0009 (5)	0.0019 (5)	0.0024 (5)
C11	0.0144 (6)	0.0186 (7)	0.0123 (7)	−0.0007 (5)	−0.0006 (5)	0.0001 (5)
C12	0.0179 (7)	0.0175 (7)	0.0143 (7)	−0.0001 (5)	0.0023 (6)	−0.0013 (6)
C13	0.0134 (6)	0.0167 (7)	0.0104 (6)	0.0002 (5)	0.0014 (5)	0.0008 (5)
C14	0.0134 (6)	0.0154 (7)	0.0100 (6)	−0.0005 (5)	0.0014 (5)	−0.0011 (5)
C15	0.0153 (6)	0.0152 (7)	0.0115 (6)	0.0000 (5)	0.0037 (5)	−0.0016 (5)
C16	0.0141 (6)	0.0169 (7)	0.0152 (7)	0.0014 (5)	0.0021 (5)	−0.0002 (6)
C17	0.0134 (6)	0.0181 (7)	0.0114 (7)	0.0011 (5)	0.0033 (5)	−0.0007 (5)
C18	0.0127 (6)	0.0165 (7)	0.0109 (6)	0.0004 (5)	0.0017 (5)	0.0002 (5)
C19	0.0127 (6)	0.0186 (7)	0.0174 (7)	0.0009 (5)	0.0023 (5)	0.0026 (6)
C20	0.0139 (6)	0.0156 (7)	0.0197 (8)	0.0025 (5)	0.0013 (6)	0.0013 (6)
C21	0.0202 (7)	0.0184 (8)	0.0285 (9)	−0.0009 (6)	0.0061 (7)	−0.0004 (6)
C22	0.0219 (8)	0.0226 (9)	0.0420 (11)	−0.0063 (6)	0.0067 (8)	−0.0074 (8)
C23	0.0239 (8)	0.0252 (9)	0.0322 (10)	−0.0005 (6)	−0.0007 (7)	−0.0123 (7)
C24	0.0234 (8)	0.0249 (8)	0.0220 (8)	0.0014 (6)	0.0024 (6)	−0.0053 (7)
C25	0.0128 (6)	0.0155 (7)	0.0179 (7)	0.0026 (5)	0.0013 (5)	0.0007 (6)
C27	0.0214 (8)	0.0306 (9)	0.0188 (8)	0.0040 (6)	−0.0042 (6)	−0.0030 (7)
C26	0.0169 (7)	0.0229 (8)	0.0164 (7)	0.0025 (6)	0.0010 (6)	0.0006 (6)
C28	0.0159 (7)	0.0279 (9)	0.0335 (10)	−0.0003 (6)	−0.0036 (7)	−0.0061 (7)
C29	0.0154 (7)	0.0254 (9)	0.0324 (9)	−0.0020 (6)	0.0046 (7)	−0.0014 (7)
C30	0.0160 (6)	0.0135 (7)	0.0128 (7)	0.0010 (5)	0.0024 (5)	0.0018 (5)
C31	0.0164 (7)	0.0184 (7)	0.0133 (7)	0.0008 (5)	0.0034 (5)	0.0045 (6)
C32	0.0210 (7)	0.0320 (9)	0.0160 (8)	0.0043 (6)	0.0046 (6)	0.0004 (6)
C33	0.0224 (8)	0.0481 (12)	0.0221 (9)	0.0086 (7)	0.0089 (7)	0.0017 (8)
C34	0.0159 (7)	0.0407 (11)	0.0310 (10)	0.0006 (7)	0.0078 (7)	0.0090 (8)
C35	0.0196 (7)	0.0261 (9)	0.0280 (9)	−0.0039 (6)	0.0028 (7)	0.0025 (7)
C36	0.0124 (6)	0.0168 (7)	0.0143 (7)	0.0014 (5)	0.0026 (5)	−0.0011 (5)
C37	0.0206 (7)	0.0183 (7)	0.0115 (7)	0.0004 (6)	0.0011 (6)	−0.0032 (6)
C38	0.0207 (7)	0.0200 (8)	0.0117 (7)	0.0018 (6)	0.0028 (6)	0.0008 (6)
C39	0.0151 (6)	0.0171 (7)	0.0160 (7)	0.0018 (5)	0.0039 (5)	0.0011 (6)
C40	0.0204 (7)	0.0207 (8)	0.0122 (7)	−0.0023 (6)	0.0030 (6)	−0.0037 (6)
C41	0.0196 (7)	0.0208 (7)	0.0099 (7)	0.0005 (5)	0.0037 (5)	0.0001 (6)
C42	0.0176 (7)	0.0190 (7)	0.0135 (7)	0.0007 (5)	0.0000 (5)	0.0027 (6)
C43	0.0212 (7)	0.0218 (8)	0.0160 (7)	0.0015 (6)	0.0025 (6)	0.0013 (6)
C44	0.0189 (7)	0.0282 (9)	0.0180 (8)	0.0002 (6)	0.0031 (6)	0.0062 (6)
C45	0.0204 (7)	0.0199 (8)	0.0220 (8)	−0.0030 (6)	−0.0021 (6)	0.0069 (6)
C46	0.0251 (8)	0.0177 (8)	0.0189 (8)	0.0008 (6)	−0.0010 (6)	0.0011 (6)
C47	0.0204 (7)	0.0201 (8)	0.0141 (7)	0.0003 (6)	0.0023 (6)	0.0003 (6)
O4	0.0504 (9)	0.0694 (11)	0.0265 (7)	0.0151 (8)	0.0144 (7)	0.0056 (7)
C49	0.0361 (10)	0.0337 (10)	0.0349 (11)	−0.0113 (8)	0.0095 (8)	−0.0092 (8)
C50	0.0365 (10)	0.0340 (10)	0.0259 (9)	−0.0069 (8)	0.0077 (8)	−0.0023 (8)
C51	0.0412 (11)	0.0467 (12)	0.0274 (10)	−0.0031 (9)	0.0041 (8)	0.0067 (9)
C48	0.0339 (10)	0.0286 (10)	0.0622 (15)	0.0001 (8)	0.0059 (10)	0.0028 (10)

### Geometric parameters (Å, º)

**Table:**

O1—C17	1.4264 (17)	C23—C24	1.390 (2)
O1—H1O1	0.90 (2)	C23—H23A	0.9300
O2—C19	1.2167 (18)	C24—H24A	0.9300
O3—C30	1.2230 (17)	C25—C26	1.395 (2)
N1—C29	1.342 (2)	C27—C26	1.385 (2)
N1—C25	1.3421 (18)	C27—C28	1.388 (2)
N2—C24	1.337 (2)	C27—H27A	0.9300
N2—C20	1.3437 (19)	C26—H26A	0.9300
N3—C35	1.338 (2)	C28—C29	1.380 (2)
N3—C31	1.341 (2)	C28—H28A	0.9300
C1—C2	1.389 (2)	C29—H29A	0.9300
C1—C6	1.400 (2)	C30—C31	1.5062 (19)
C1—H1A	0.9300	C31—C32	1.393 (2)
C2—C3	1.385 (2)	C32—C33	1.384 (2)
C2—H2A	0.9300	C32—H32A	0.9300
C3—C4	1.388 (2)	C33—C34	1.384 (3)
C3—H3A	0.9300	C33—H33A	0.9300
C4—C5	1.393 (2)	C34—C35	1.384 (2)
C4—H4A	0.9300	C34—H34A	0.9300
C5—C6	1.399 (2)	C35—H35A	0.9300
C5—H5A	0.9300	C36—C37	1.394 (2)
C6—C7	1.4868 (19)	C36—C41	1.397 (2)
C7—C12	1.394 (2)	C37—C38	1.390 (2)
C7—C8	1.399 (2)	C37—H37A	0.9300
C8—C9	1.387 (2)	C38—C39	1.392 (2)
C8—H8A	0.9300	C38—H38A	0.9300
C9—C10	1.398 (2)	C39—C40	1.402 (2)
C9—H9A	0.9300	C39—C42	1.489 (2)
C10—C11	1.395 (2)	C40—C41	1.383 (2)
C10—C13	1.5203 (19)	C40—H40A	0.9300
C11—C12	1.391 (2)	C41—H41A	0.9300
C11—H11A	0.9300	C42—C47	1.396 (2)
C12—H12A	0.9300	C42—C43	1.401 (2)
C13—C18	1.532 (2)	C43—C44	1.384 (2)
C13—C14	1.5581 (19)	C43—H43A	0.9300
C13—H13A	0.9800	C44—C45	1.386 (2)
C14—C30	1.5207 (19)	C44—H44A	0.9300
C14—C15	1.5526 (19)	C45—C46	1.394 (2)
C14—H14A	0.9800	C45—H45A	0.9300
C15—C36	1.515 (2)	C46—C47	1.390 (2)
C15—C16	1.530 (2)	C46—H46A	0.9300
C15—H15A	0.9800	C47—H47A	0.9300
C16—C17	1.526 (2)	O4—C50	1.229 (2)
C16—H16A	0.9700	C49—C50	1.495 (3)
C16—H16B	0.9700	C49—C48	1.515 (3)
C17—C25	1.523 (2)	C49—H49A	0.9700
C17—C18	1.5738 (19)	C49—H49B	0.9700
C18—C19	1.517 (2)	C50—C51	1.500 (3)
C18—H18A	0.9800	C51—H51A	0.9600
C19—C20	1.510 (2)	C51—H51B	0.9600
C20—C21	1.393 (2)	C51—H51C	0.9600
C21—C22	1.382 (2)	C48—H48A	0.9600
C21—H21A	0.9300	C48—H48B	0.9600
C22—C23	1.385 (3)	C48—H48C	0.9600
C22—H22A	0.9300		
			
C17—O1—H1O1	104.1 (14)	N2—C24—H24A	118.4
C29—N1—C25	118.00 (14)	C23—C24—H24A	118.4
C24—N2—C20	117.38 (13)	N1—C25—C26	122.42 (14)
C35—N3—C31	116.27 (14)	N1—C25—C17	114.38 (13)
C2—C1—C6	120.88 (15)	C26—C25—C17	123.10 (13)
C2—C1—H1A	119.6	C26—C27—C28	118.76 (16)
C6—C1—H1A	119.6	C26—C27—H27A	120.6
C3—C2—C1	120.33 (15)	C28—C27—H27A	120.6
C3—C2—H2A	119.8	C27—C26—C25	118.82 (14)
C1—C2—H2A	119.8	C27—C26—H26A	120.6
C2—C3—C4	119.45 (14)	C25—C26—H26A	120.6
C2—C3—H3A	120.3	C29—C28—C27	118.86 (15)
C4—C3—H3A	120.3	C29—C28—H28A	120.6
C3—C4—C5	120.56 (15)	C27—C28—H28A	120.6
C3—C4—H4A	119.7	N1—C29—C28	123.07 (15)
C5—C4—H4A	119.7	N1—C29—H29A	118.5
C4—C5—C6	120.44 (14)	C28—C29—H29A	118.5
C4—C5—H5A	119.8	O3—C30—C31	118.39 (13)
C6—C5—H5A	119.8	O3—C30—C14	122.63 (12)
C5—C6—C1	118.34 (14)	C31—C30—C14	118.87 (12)
C5—C6—C7	121.81 (13)	N3—C31—C32	123.92 (14)
C1—C6—C7	119.83 (14)	N3—C31—C30	117.80 (13)
C12—C7—C8	118.01 (13)	C32—C31—C30	118.25 (14)
C12—C7—C6	120.86 (13)	C33—C32—C31	118.28 (16)
C8—C7—C6	121.11 (13)	C33—C32—H32A	120.9
C9—C8—C7	120.64 (14)	C31—C32—H32A	120.9
C9—C8—H8A	119.7	C32—C33—C34	118.78 (16)
C7—C8—H8A	119.7	C32—C33—H33A	120.6
C8—C9—C10	121.30 (14)	C34—C33—H33A	120.6
C8—C9—H9A	119.3	C33—C34—C35	118.46 (15)
C10—C9—H9A	119.3	C33—C34—H34A	120.8
C11—C10—C9	117.98 (13)	C35—C34—H34A	120.8
C11—C10—C13	123.31 (13)	N3—C35—C34	124.23 (16)
C9—C10—C13	118.49 (13)	N3—C35—H35A	117.9
C12—C11—C10	120.69 (14)	C34—C35—H35A	117.9
C12—C11—H11A	119.7	C37—C36—C41	117.88 (14)
C10—C11—H11A	119.7	C37—C36—C15	122.96 (13)
C11—C12—C7	121.28 (14)	C41—C36—C15	119.11 (13)
C11—C12—H12A	119.4	C38—C37—C36	121.04 (14)
C7—C12—H12A	119.4	C38—C37—H37A	119.5
C10—C13—C18	114.64 (12)	C36—C37—H37A	119.5
C10—C13—C14	109.00 (11)	C37—C38—C39	121.02 (14)
C18—C13—C14	110.86 (11)	C37—C38—H38A	119.5
C10—C13—H13A	107.3	C39—C38—H38A	119.5
C18—C13—H13A	107.3	C38—C39—C40	117.84 (14)
C14—C13—H13A	107.3	C38—C39—C42	122.19 (13)
C30—C14—C15	114.84 (12)	C40—C39—C42	119.86 (13)
C30—C14—C13	108.39 (11)	C41—C40—C39	121.01 (14)
C15—C14—C13	110.57 (11)	C41—C40—H40A	119.5
C30—C14—H14A	107.6	C39—C40—H40A	119.5
C15—C14—H14A	107.6	C40—C41—C36	121.10 (14)
C13—C14—H14A	107.6	C40—C41—H41A	119.5
C36—C15—C16	114.71 (12)	C36—C41—H41A	119.5
C36—C15—C14	110.65 (11)	C47—C42—C43	118.11 (14)
C16—C15—C14	112.15 (12)	C47—C42—C39	121.60 (13)
C36—C15—H15A	106.2	C43—C42—C39	120.12 (14)
C16—C15—H15A	106.2	C44—C43—C42	120.60 (15)
C14—C15—H15A	106.2	C44—C43—H43A	119.7
C17—C16—C15	111.45 (12)	C42—C43—H43A	119.7
C17—C16—H16A	109.3	C43—C44—C45	120.87 (14)
C15—C16—H16A	109.3	C43—C44—H44A	119.6
C17—C16—H16B	109.3	C45—C44—H44A	119.6
C15—C16—H16B	109.3	C44—C45—C46	119.23 (14)
H16A—C16—H16B	108.0	C44—C45—H45A	120.4
O1—C17—C25	109.25 (11)	C46—C45—H45A	120.4
O1—C17—C16	107.96 (12)	C47—C46—C45	119.90 (15)
C25—C17—C16	111.72 (12)	C47—C46—H46A	120.1
O1—C17—C18	109.20 (11)	C45—C46—H46A	120.1
C25—C17—C18	109.53 (11)	C46—C47—C42	121.21 (14)
C16—C17—C18	109.14 (11)	C46—C47—H47A	119.4
C19—C18—C13	112.49 (12)	C42—C47—H47A	119.4
C19—C18—C17	108.47 (11)	C50—C49—C48	113.78 (17)
C13—C18—C17	109.89 (11)	C50—C49—H49A	108.8
C19—C18—H18A	108.6	C48—C49—H49A	108.8
C13—C18—H18A	108.6	C50—C49—H49B	108.8
C17—C18—H18A	108.6	C48—C49—H49B	108.8
O2—C19—C20	119.92 (14)	H49A—C49—H49B	107.7
O2—C19—C18	122.87 (14)	O4—C50—C49	121.41 (19)
C20—C19—C18	117.14 (12)	O4—C50—C51	121.67 (18)
N2—C20—C21	123.16 (15)	C49—C50—C51	116.93 (16)
N2—C20—C19	117.26 (13)	C50—C51—H51A	109.5
C21—C20—C19	119.58 (14)	C50—C51—H51B	109.5
C22—C21—C20	118.56 (15)	H51A—C51—H51B	109.5
C22—C21—H21A	120.7	C50—C51—H51C	109.5
C20—C21—H21A	120.7	H51A—C51—H51C	109.5
C21—C22—C23	118.87 (15)	H51B—C51—H51C	109.5
C21—C22—H22A	120.6	C49—C48—H48A	109.5
C23—C22—H22A	120.6	C49—C48—H48B	109.5
C22—C23—C24	118.73 (16)	H48A—C48—H48B	109.5
C22—C23—H23A	120.6	C49—C48—H48C	109.5
C24—C23—H23A	120.6	H48A—C48—H48C	109.5
N2—C24—C23	123.24 (16)	H48B—C48—H48C	109.5
			
C6—C1—C2—C3	0.7 (2)	C21—C22—C23—C24	−1.7 (3)
C1—C2—C3—C4	−0.7 (2)	C20—N2—C24—C23	−1.6 (2)
C2—C3—C4—C5	0.1 (2)	C22—C23—C24—N2	2.8 (3)
C3—C4—C5—C6	0.4 (2)	C29—N1—C25—C26	−1.9 (2)
C4—C5—C6—C1	−0.4 (2)	C29—N1—C25—C17	174.52 (13)
C4—C5—C6—C7	177.71 (14)	O1—C17—C25—N1	11.29 (17)
C2—C1—C6—C5	−0.2 (2)	C16—C17—C25—N1	130.67 (13)
C2—C1—C6—C7	−178.30 (15)	C18—C17—C25—N1	−108.28 (14)
C5—C6—C7—C12	146.89 (15)	O1—C17—C25—C26	−172.28 (13)
C1—C6—C7—C12	−35.1 (2)	C16—C17—C25—C26	−52.89 (18)
C5—C6—C7—C8	−35.0 (2)	C18—C17—C25—C26	68.15 (17)
C1—C6—C7—C8	143.08 (15)	C28—C27—C26—C25	−0.2 (2)
C12—C7—C8—C9	2.4 (2)	N1—C25—C26—C27	2.2 (2)
C6—C7—C8—C9	−175.83 (14)	C17—C25—C26—C27	−173.98 (14)
C7—C8—C9—C10	0.2 (2)	C26—C27—C28—C29	−1.8 (2)
C8—C9—C10—C11	−2.6 (2)	C25—N1—C29—C28	−0.2 (2)
C8—C9—C10—C13	172.17 (13)	C27—C28—C29—N1	2.1 (3)
C9—C10—C11—C12	2.4 (2)	C15—C14—C30—O3	51.17 (19)
C13—C10—C11—C12	−172.06 (13)	C13—C14—C30—O3	−73.02 (17)
C10—C11—C12—C7	0.1 (2)	C15—C14—C30—C31	−132.65 (13)
C8—C7—C12—C11	−2.5 (2)	C13—C14—C30—C31	103.16 (14)
C6—C7—C12—C11	175.67 (14)	C35—N3—C31—C32	−1.4 (2)
C11—C10—C13—C18	−23.20 (19)	C35—N3—C31—C30	176.63 (14)
C9—C10—C13—C18	162.34 (12)	O3—C30—C31—N3	−152.71 (14)
C11—C10—C13—C14	101.71 (15)	C14—C30—C31—N3	30.95 (19)
C9—C10—C13—C14	−72.76 (16)	O3—C30—C31—C32	25.5 (2)
C10—C13—C14—C30	−55.26 (15)	C14—C30—C31—C32	−150.88 (14)
C18—C13—C14—C30	71.82 (14)	N3—C31—C32—C33	−0.4 (3)
C10—C13—C14—C15	178.04 (11)	C30—C31—C32—C33	−178.48 (15)
C18—C13—C14—C15	−54.88 (15)	C31—C32—C33—C34	1.8 (3)
C30—C14—C15—C36	59.49 (16)	C32—C33—C34—C35	−1.3 (3)
C13—C14—C15—C36	−177.48 (11)	C31—N3—C35—C34	2.0 (2)
C30—C14—C15—C16	−69.98 (15)	C33—C34—C35—N3	−0.7 (3)
C13—C14—C15—C16	53.05 (15)	C16—C15—C36—C37	43.43 (18)
C36—C15—C16—C17	176.73 (11)	C14—C15—C36—C37	−84.67 (16)
C14—C15—C16—C17	−55.93 (15)	C16—C15—C36—C41	−139.07 (13)
C15—C16—C17—O1	−60.21 (14)	C14—C15—C36—C41	92.83 (15)
C15—C16—C17—C25	179.66 (11)	C41—C36—C37—C38	−3.4 (2)
C15—C16—C17—C18	58.38 (15)	C15—C36—C37—C38	174.09 (13)
C10—C13—C18—C19	−56.49 (15)	C36—C37—C38—C39	1.7 (2)
C14—C13—C18—C19	179.59 (11)	C37—C38—C39—C40	1.4 (2)
C10—C13—C18—C17	−177.45 (11)	C37—C38—C39—C42	−174.85 (13)
C14—C13—C18—C17	58.63 (14)	C38—C39—C40—C41	−2.8 (2)
O1—C17—C18—C19	−65.58 (14)	C42—C39—C40—C41	173.55 (14)
C25—C17—C18—C19	54.02 (15)	C39—C40—C41—C36	1.1 (2)
C16—C17—C18—C19	176.62 (12)	C37—C36—C41—C40	2.1 (2)
O1—C17—C18—C13	57.77 (14)	C15—C36—C41—C40	−175.58 (13)
C25—C17—C18—C13	177.36 (11)	C38—C39—C42—C47	−140.90 (15)
C16—C17—C18—C13	−60.04 (15)	C40—C39—C42—C47	42.9 (2)
C13—C18—C19—O2	−36.79 (19)	C38—C39—C42—C43	43.9 (2)
C17—C18—C19—O2	84.98 (17)	C40—C39—C42—C43	−132.31 (15)
C13—C18—C19—C20	146.37 (12)	C47—C42—C43—C44	−3.0 (2)
C17—C18—C19—C20	−91.86 (14)	C39—C42—C43—C44	172.36 (14)
C24—N2—C20—C21	−0.5 (2)	C42—C43—C44—C45	1.0 (2)
C24—N2—C20—C19	−179.92 (13)	C43—C44—C45—C46	1.4 (2)
O2—C19—C20—N2	150.38 (14)	C44—C45—C46—C47	−1.8 (2)
C18—C19—C20—N2	−32.68 (18)	C45—C46—C47—C42	−0.3 (2)
O2—C19—C20—C21	−29.0 (2)	C43—C42—C47—C46	2.7 (2)
C18—C19—C20—C21	147.93 (14)	C39—C42—C47—C46	−172.64 (14)
N2—C20—C21—C22	1.5 (2)	C48—C49—C50—O4	7.2 (3)
C19—C20—C21—C22	−179.16 (14)	C48—C49—C50—C51	−172.71 (17)
C20—C21—C22—C23	−0.3 (2)		

### Hydrogen-bond geometry (Å, º)

Cg2 is the centroid of the N2/C20–C24 ring.

**Table:**

*D*—H···*A*	*D*—H	H···*A*	*D*···*A*	*D*—H···*A*
O1—H1*O*1···N1	0.90 (2)	1.92 (2)	2.5615 (18)	127.0 (19)
C16—H16*A*···O3	0.97	2.46	3.0730 (19)	121
C18—H18*A*···O3	0.98	2.39	3.0603 (19)	125
C26—H26*A*···O1^i^	0.93	2.53	3.3079 (19)	142
C27—H27*A*···O4^ii^	0.93	2.56	3.464 (2)	163
C3—H3*A*···*Cg*2^iii^	0.93	2.96	3.7821 (17)	148

Symmetry codes: (i) *x*, −*y*+1/2, *z*+1/2; (ii) −*x*+1, −*y*+1, −*z*+1; (iii) −*x*, −*y*+1, −*z*.
